# Development of Robust Steel Alloys for Laser-Directed Energy Deposition via Analysis of Mechanical Property Sensitivities †

**DOI:** 10.3390/mi15101180

**Published:** 2024-09-24

**Authors:** Jonathan Kelley, Joseph W. Newkirk, Laura N. Bartlett, Sriram Praneeth Isanaka, Todd Sparks, Saeid Alipour, Frank Liou

**Affiliations:** 1 Department of Mechanical and Aerospace Engineering, Missouri University of Science and Technology, Rolla, MO 65409, USA; sihyd@mst.edu; 2 Department of Nuclear Engineering and Radiation Science, Missouri University of Science and Technology, Rolla, MO 65409, USA; jnewkirk@mst.edu; 3 Department of Materials Science and Engineering, Missouri University of Science and Technology, Rolla, MO 65409, USA; lnmkvf@mst.edu (L.N.B.); saeid.alipour@mst.edu (S.A.); 4 Product Innovation and Engineering LLC, St. James, MO 65559, USA; toddesparks@mopine.com

**Keywords:** laser-directed energy deposition, alloy design, robustness, sensitivity analysis, high-strength low-alloy steel, in situ alloying, powder blend

## Abstract

To ensure consistent performance of additively manufactured metal parts, it is advantageous to identify alloys that are robust to process variations. This paper investigates the effect of steel alloy composition on mechanical property robustness in laser-directed energy deposition (L-DED). In situ blending of ultra-high-strength low-alloy steel (UHSLA) and pure iron powders produced 10 compositions containing 10–100 wt% UHSLA. Samples were deposited using a novel configuration that enabled rapid collection of hardness data. The Vickers hardness sensitivity of each alloy was evaluated with respect to laser power and interlayer delay time. Yield strength (YS) and ultimate tensile strength (UTS) sensitivities of five select alloys were investigated in a subsequent experiment. Microstructure analysis revealed that cooling rate-driven phase fluctuations between lath martensite and upper bainite were a key factor leading to high hardness sensitivity. By keeping the UHSLA content ≤20% or ≥70%, the microstructure transformed primarily to ferrite or martensite, respectively, which generally corresponded to improved robustness. Above 70% UHSLA, the YS sensitivity remained low while the UTS sensitivity increased. This finding, coupled with the observation of auto-tempered martensite at lower cooling rates, may suggest a strong response of the work hardening capability to auto-tempering at higher alloy contents. This work demonstrates a methodology for incorporating robust design into the development of alloys for additive manufacturing.

## 1. Introduction

Additive manufacturing (AM), commonly known as 3D printing, has emerged in recent decades as a pivotal technology enabling the rapid fabrication of geometrically complex parts. In AM, components are built in a layer-by-layer fashion directly from a digital 3D model. Three of the most common AM processes for metals are laser-powder bed fusion (L-PBF), laser-directed energy deposition (L-DED), and wire-arc AM (WAAM). In L-PBF, each layer of the part is defined by selectively melting a region within a thin layer of metal powder spread onto a base plate [1]. In L-DED, a metal feedstock in wire or powder form is injected through a nozzle into a melt pool generated by a laser [2]. In WAAM, an electric arc is used to melt a wire feedstock [3]. Metal AM enables rapid low-quantity production while minimizing material waste and avoiding the need for specialized tooling, with applications often found in aerospace, biomedical, and energy sectors [4].

While offering many advantages, metal AM comes with substantial challenges owing to the rapid cooling rates, complex thermal cycles, and melt pool dynamics inherent to the process, which are similar to those occurring in multi-pass welding [5,6]. Under such conditions, many alloys are highly susceptible to defects including porosity, distortion, and cracking [7]. Process parameters such as laser power and scan speed, as well as part size and geometry, work together to influence the thermal history, which, in turn, influences the microstructure and mechanical properties of the consolidated material [8]. Cooling rates can vary significantly among AM processes, with typical rates being on the order of 10^4^–10^6^ °C/s in L-PBF [9], 10^3^–10^4^ °C/s in L-DED [8], and 100 °C/s in WAAM [3].

Many research efforts have investigated the AM of existing alloys such as 316L stainless steel, Ti-6Al-4V, and Inconel 718 [10]. By comparison, relatively few have aimed at developing alloys tailored toward AM. Within the alloy design space, various studies have focused on improving printability, i.e., defect resistance [11,12,13,14]. Others have investigated alloy modifications to promote equiaxed grain structures [15,16]. One advantage offered by L-DED is the ability to explore new compositions via the in situ blending of powders. Several studies have leveraged this feature for rapid alloy development [12,17,18,19,20]. Once a desirable feedstock composition is identified, it can be pre-alloyed for use in industrial AM [21]. Integrated computational materials engineering (ICME), thermodynamic modeling, and machine learning have also been used for alloy design [7,12,19,22,23].

**Figure 1:**
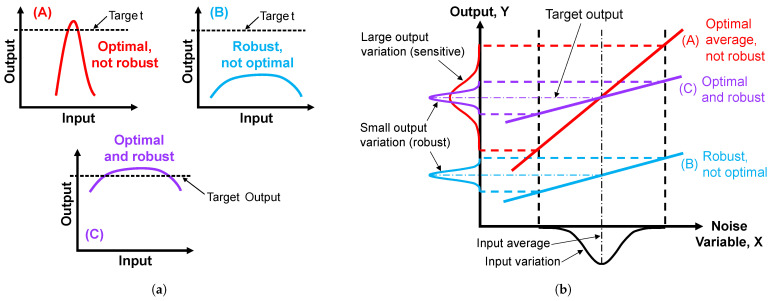
Distinction between robust and optimal: (**a**) arbitrary function and (**b**) linear system.

In order for metal AM to be a viable solution, it must be able to consistently produce parts that meet performance criteria. To this end, robust design strategies can be employed. A robust system is one whose output is relatively insensitive to noise variables [24]. In a given system, control factors are those which can be reliably specified, while noise factors are uncertain and difficult to control. Robust design (Type 1) seeks to minimize the effects of noise through the tuning of control factors [25]. Importantly, good robustness does not necessarily imply optimal performance. With reference to Figure 1a, System A (red) is highly sensitive to noise inputs; therefore, it only achieves target performance under a narrow set of conditions. System B (blue) is robust but not optimal, while System C (purple) is both optimal and robust. For a linear system, improved robustness corresponds to a shallower slope of the input–output relationship, as illustrated in Figure 1b.

Sagar et al. [26] and Hu et al. [27] highlighted opportunities to apply robust design to the improvement of AM processes. Outputs of interest may include melt pool characteristics, defect content, microstructures, mechanical properties, etc. Control factors may include parameters such as laser power and scan speed, while noise factors may include particle size distribution [26] or variations in the absorptivity of the metal powder [27]. Machine learning techniques [28,29,30], finite element models [31], and response surface methods [32] have been used to identify robust operating windows and/or determine which inputs exert the strongest influence on the output of interest. Stavropoulos et al. presented a digital twin framework for robust AM process control [33].

Alternatively, robust design can focus on the development of the feedstock alloy, with the alloying elements serving as control factors. Haines et al. studied the effect of alloy composition on the columnar-to-equiaxed transition (CET) of Ni-based superalloys with the goal of promoting equiaxed grain structures across a range of process conditions [16]. Johnson et al. developed printability maps for two alloys, proposing the size of the printable processing window as a metric for robustness [13]. Using a reliability-based approach, Wang et al. employed an ICME framework to design an optimized variation of HSLA-115, a high-strength low-alloy (HSLA) steel, under compositional uncertainty [22].

HSLA steels, which are widely used in structural applications such as pipelines, naval vessels, and automotive components, have shown promise in AM for direct fabrication and part repair [34]. For example, Wang et al. developed an underwater L-DED process for the offshore repair of HSLA-100 marine equipment [35,36]. Numerous microstructural factors can influence the mechanical properties of steels [37]. Depending on the composition and cooling rate, HSLA steel microstructures may consist of polygonal ferrite, acicular ferrite, pearlite, bainite, martensite, retained austenite, or a mixture of phases [38,39,40]. As the carbon equivalent (CE) value increases, hardenability increases, meaning martensite transformation occurs more readily [41,42]. Alloy robustness is especially critical in repair, as it is difficult to tightly control the thermal history when adapting to a unique repair surface. Acknowledging this, Barr et al. [43] and Wang et al. [44] compared multiple steel feedstocks to determine which compositions were more conducive to repair.

Aiming to fill a gap in the current literature, the present work systematically investigates mechanical property sensitivities in powder-based L-DED over a wide composition space to identify steel alloys that are robust to process variations. A two-feeder system blended ultra-high-strength low-alloy steel (UHSLA) and pure iron powders in situ, producing 10 compositions containing 10–100 wt% UHSLA. In this manner, the composition space encompassed alloys ranging from low to high hardenability, with carbon equivalent values between roughly 0.135 wt% and 1.35 wt% (Table 1 and ([41], p. 2, Eq. 2)). This ensured a diverse array of microstructural phases could be examined. In Experiment #1, thin-wall samples were deposited using a novel setup that enabled rapid collection of hardness data. The Vickers hardness sensitivity of each alloy was evaluated with respect to laser power and interlayer delay time via multiple linear regression. In Experiment #2, five select alloys were deposited, and their tensile property sensitivities were evaluated with respect to interlayer delay. Microstructural phases were analyzed to aid the interpretation of the results. The process parameters were treated as noise factors, while the alloy composition was the control factor. Laser power is a common parameter that can vary among AM systems. “Interlayer delay” refers to a pause during which the laser is switched off between layers. Variation of this input is intended to mimic a prevalent noise factor in complex parts for which the laser return time varies at different locations within the build.

Note: This article is an extended version of a paper entitled “Influence of steel alloy composition on the process robustness of as-built hardness in laser-directed energy deposition”, which was presented at the 34th Annual International Solid Freeform Fabrication Symposium held in Austin, TX, USA, 14–16 August 2023 [45].

## 2. Materials and Methods

### 2.1. Quantifying Robustness

As illustrated in Figure 1, a more robust system results in less output variability. While the standard deviation is a simple metric for variability, it does not provide causal insight. To assess an output’s response to a set of input factors, a design of experiments (DOE) methodology coupled with multiple regression can be employed. If the input–output correlation is linear, the regression model is of the following form:

(1) $$y=\beta_{0}+\beta_{1}x_{1}+\beta_{2}x_{2}+\ldots +\beta_{n}x_{n}$$

In Equation (1), *y* represents the output variable, $\beta_{0}$ represents the *y*-intercept, $x_{1}$–$x_{n}$ represent the *n* input variables, and $\beta_{1}$–$\beta_{n}$ represent the coefficients corresponding to the input variables. A given coefficient $\beta_{i}$ represents the slope of the input–output relationship with respect to the input variable $x_{i}$ with all other inputs held constant [46]. A few important metrics are used to evaluate the regression model. The coefficient of determination R^2^ indicates the proportion of the total output variation that is explained by the model as a whole, with R^2^ closer to 1 indicating a better fit ([47], pp. 52–54). The *p*-value corresponding to each input variable indicates whether that variable has a statistically significant effect on the output. The LogWorth, which is equal to −log_10_(*p*-value), is sometimes used in place of the *p*-value for ease of graphical comparisons. Higher LogWorth (lower *p*-value) indicates higher statistical significance. A LogWorth > 1.3, which corresponds to a *p*-value < 0.05, is required for an effect to be considered statistically significant to a level α=0.05 ([47], pp. 66–81). Importantly, high statistical significance does not necessarily imply practical significance. The practical significance of an input variable is indicated by the slope of the output’s response to that variable as quantified by the $\beta_{i}$ coefficient.

In Experiment #1 of this study, *y* is the Vickers hardness (HV), $x_{1}$ is the laser power (LP) measured in watts (W), and $x_{2}$ is the interlayer delay (ID) measured in seconds (s). Thus, the linear regression model can be written as follows:

(2) $$HV=\beta_{0}+\beta_{LP}LP+\beta_{ID}ID$$

**Figure 2:**
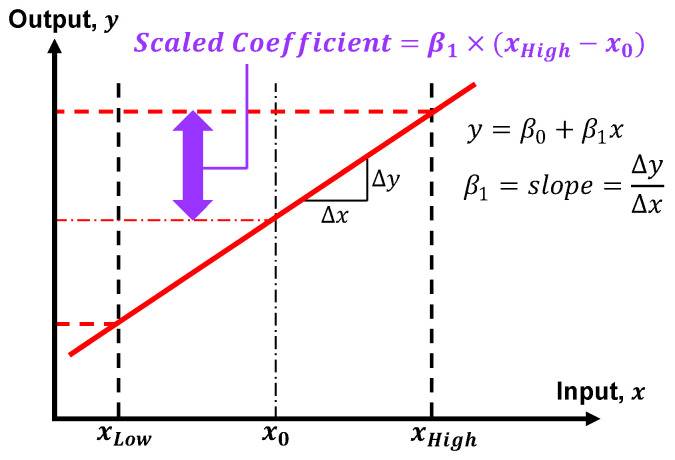
Calculation of the scaled coefficient illustrated for a single-input linear system.

Since the two input variables have different units associated with them, $\beta_{LP}$ and $\beta_{ID}$ must be scaled before their magnitudes can be compared directly. Scaled coefficients (SCs) can be obtained by multiplying each $\beta_{i}$ coefficient by 1/2 the experimental range of its corresponding input variable $x_{i}$. This is illustrated in Figure 2 for a single-input system, where $x_{Low}$, $x_{High}$, and $x_{0}$ represent the minimum, maximum, and midpoint input levels employed in the experiment, respectively. The SC measures the amount of change in the output as a specified input travels from its midpoint $x_{0}$ to either end of its experimental sample space, with all other inputs held constant [48]. In Experiment #1, the laser power varied from 650 W to 1050 W (range/2 = 200 W), and the interlayer delay varied from 1 s to 11 s (range/2 = 5 s). Thus, the scaled coefficients are $SC_{LP}=\beta_{LP}\times \left(200\;W\right)$ and $SC_{ID}=\beta_{ID}\times \left(5\;s\right)$, where $SC_{LP}$ and $SC_{ID}$ have units of Vickers hardness (HV).

In Experiment #2, *y* is a tensile property (e.g., yield strength). The ID was again varied from 1 s to 11 s, while the LP was held constant (see beginning of Section 2.4 for explanation). In this case, $SC_{ID}$ has units corresponding to the tensile property at hand.

The quantities $|SC_{LP}|$ and $|SC_{ID}|$, along with the standard deviation, were the key metrics used to assess the robustness of the deposited alloys. First, the R^2^ and LogWorth metrics were used to determine how well the regression models fit the data and to discover whether statistically significant correlations existed. Next, the SCs were analyzed. If alloy A exhibited a smaller response than alloy B as indicated by a decrease in $|SC_{LP}|$ and $|SC_{ID}|$, and if this decrease was accompanied by a reduction in the overall variability as indicated by the standard deviation, then alloy A was considered to be more robust than alloy B.

### 2.2. Materials and Equipment

**Figure 3:**
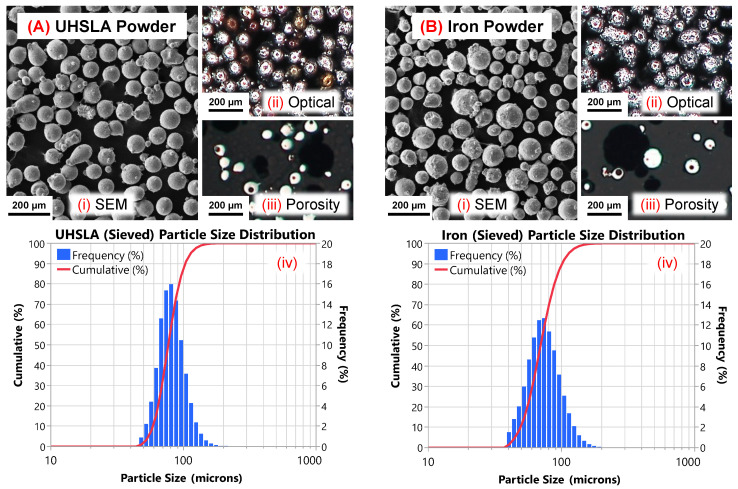
(**A**) UHSLA powder and (**B**) iron powder: (**i**) SEM, (**ii**) optical, (**iii**) porosity, and (**iv**) PSD.

UHSLA and pure iron powders (gas-atomized) were purchased from Powder Alloy Corporation (PAC) and Atlantic Equipment Engineers (AEE), respectively. The chemical compositions, as reported by the suppliers, are provided in Table 1. The as-received UHSLA powder was sieved to obtain a particle size range of ∼75–106 μm. The iron powder was sieved to remove particles below 53 μm. Three static light scattering (SLS) measurements were averaged to obtain the particle size distributions (PSDs) of the powders. Figure 3 provides scanning electron microscope (SEM) images, optical images, and PSDs of the two powders after sieving. Note the surface oxidation on the UHSLA powder and porosity inside both powders. The average D10/D50/D90 after sieving was 59.0/76.8/103.2 μm for UHSLA and 49.3/69.6/100.9 μm for the pure iron powder. The similar PSDs help promote equal capture of both powders in the melt pool, as particle size can have a substantial influence on catchment efficiency [49]. The enthalpy of mixing between Fe and each of the major alloying elements in UHSLA (C, Cr, Ni, Si, Mo, Mn, and V) is negative or zero [50], indicating conditions are thermodynamically favorable for the in situ formation of a homogeneous alloy [51].

**Figure 4:**
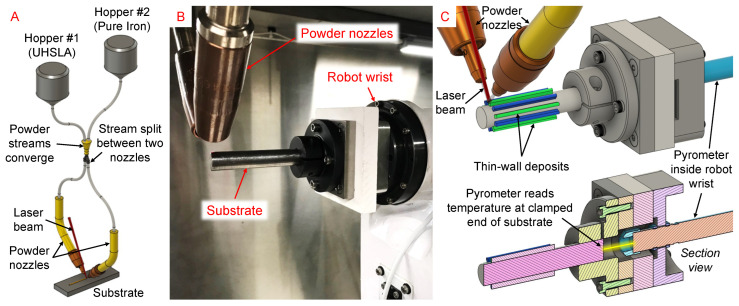
Experiment #1 L-DED setup: (**A**) powder delivery, (**B**) rotary system photo, (**C**) 3D model.

**Figure 5:**
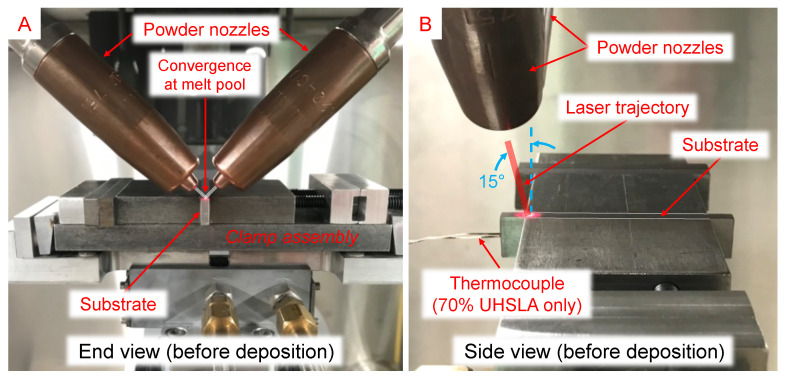
Experiment #2 L-DED setup: (**A**) end view photo and (**B**) side view photo.

The L-DED system depicted in Figure 4 used a TeraDiode DLS-0970-02000-DBS direct-diode laser (wavelength 978 nm) with 2-kW maximum output power, tilted 15° from vertical. A Nachi MZ07 6-axis robot controlled the movement of the substrate. The UHSLA and pure iron powders were metered from an electrostatically-cleared wheel feeder and vibrational feeder, respectively, both manufactured by Powder Motion Labs. The two powder lines converged downstream to achieve in situ mixing. The blended stream was then split between two nozzles before intersecting the laser beam at the melt pool (Figure 4A). In Experiment #1, a novel rotary setup was used in which thin-wall samples were deposited around the circumference of a cylindrical substrate clamped by the robot (Figure 4B,C). This allowed multiple deposits to fit inside a small space, minimizing the time required for mounting and polishing. A pyrometer inside the robot wrist monitored the temperature of the substrate’s clamped end. In Experiment #2, individual thin-wall samples were deposited on separate substrates cut from rectangular bar stock, as depicted in Figure 5.

**Table 1:** Chemical composition (wt%) of the metal powders.

Element	Fe	Cr	Ni	Si	Mo	Mn	C	V	Cu
**UHSLA powder**	Balance	2.6	1.0	1.0	0.86	0.6	0.28	0.15	<0.2
**Iron powder**	99.89% min	0.0295	0.008	0.0001	0.001	0.012	0.0075	-	0.0095
**Element**	**O**	**P**	**S**	**Al**	**Ti**	**P+Sn+As+Sb**	**H**	**N**	**W**
**UHSLA powder**	0.03	0.009	0.005	0.004	<0.006	<0.035	6 ppm	-	-
**Iron powder**	431 ppm	-	-	-	0.0038	-	-	63 ppm	0.042

### 2.3. Experiment #1: Hardness Sensitivity

**Figure 6:**
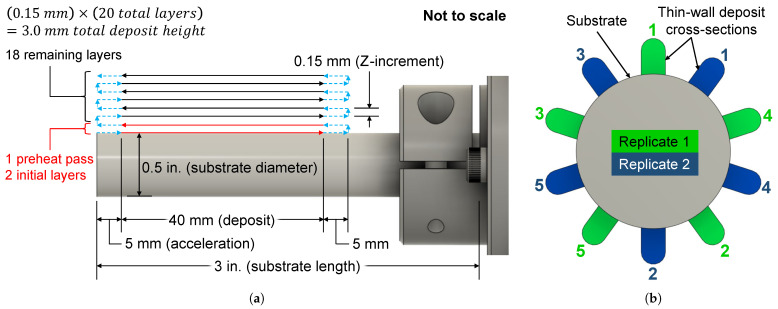
Deposition strategy for each alloy: (**a**) side view and (**b**) end view depicting the run order.

Following a design of experiments (DOE) methodology, a 2-factor, 2-level experiment (plus 1 center point) was employed to assess the impacts of laser power and interlayer delay time on the as-built hardness of 10 alloy compositions. The experiment included 2^2^ + 1 = 5 total combinations of input factors. Two replicates of the experiment, each with a different randomized run order, were carried out using a single substrate for each composition. Within a replicate, 5 thin-wall samples were deposited corresponding to the 5 factor level combinations, with a 2-min pause between samples. Before beginning replicate 2, the substrate was allowed to cool to ∼30 °C. Figure 6a illustrates the deposition strategy for each individual thin wall. As depicted in Figure 6b, samples were deposited in a star-like fashion around the circumference of the substrate, with the numbers 1–5 corresponding to the runs in Table 2. This pattern was employed in an effort to balance the heat input around the substrate. Samples in replicate 2 were deposited between those of replicate 1. The minimum laser power was specified to prevent lack of fusion defects, while the upper limit was designated to avoid excessive keyholing. Keyholing effects were additionally mitigated by depositing slightly below the laser’s focal point, which helps spread out the beam’s energy.

**Table 2:** Thin-wall rotary deposition DOE (randomized).

Replicate 1					
**Run**	**1**	**2**	**3**	**4**	**5**
**Laser Power (W)**	650	1050	850	1050	650
**Interlayer Delay (s)**	11	1	6	11	1
**Replicate 2**					
**Run**	**1**	**2**	**3**	**4**	**5**
**Laser Power (W)**	650	1050	650	850	1050
**Interlayer Delay (s)**	1	11	11	6	1

For all deposits, the scan speed was 10 mm/s, and the total powder feed rate was 2.5 g/min. The individual feed rates of the UHSLA and iron powders determined the composition, which ranged from 10–100% UHSLA in increments of 10% by mass. Argon carrier gas and shield gas were run at 0.12 L/min through each powder line and 3.0 L/min around each nozzle, respectively. The substrates consisted of 1018 low-carbon steel round stock, 3′′ long × 0.5′′ in diameter. The deposit length was 40 mm, plus a 5-mm distance on either end in which the laser was switched off while the robot decelerated and accelerated to begin the next layer, ensuring constant velocity within the deposited area. Note the interlayer delay includes the time required to travel this 5-mm distance. Twenty layers were deposited with a 0.15-mm Z-increment between each layer, giving a total height of about 3.0 mm. The wall thickness was ∼1.5–2.5 mm depending on the laser power, with higher laser powers giving wider tracks. A single preheat pass was run at a laser power of 1050 W. The first 2 layers were deposited with a laser power of 1050 W and interlayer delay of 1 s to ensure bonding with the substrate. The remaining 18 layers were deposited using parameters dictated by the experimental run in Table 2.

**Figure 7:**
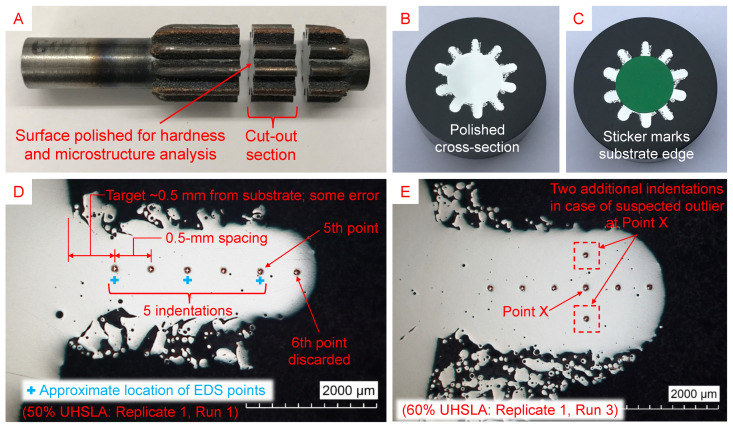
(**A**) One of the substrates containing 10 thin-wall deposits, (**B**) polished cross-section, (**C**) sticker to mark the substrate edge, (**D**) hardness indentation layout, and (**E**) extra indentations in case of a suspected outlier.

The completed experiments resulted in 10 substrates corresponding to the 10 compositions, each containing 10 deposits corresponding to the 10 experimental runs (5 runs per replicate). Figure 7A shows one of these sample-laden substrates after being sectioned for hardness and microstructure analysis, with the analyzed surface located around the middle of the deposit length. The cut-out sections were hot-mounted and polished to a 0.25-μm finish. A METPREP 3 PH-3 machine and consumables by Allied High Tech Products (Cerritos, CA, USA) were used for grinding and polishing. The grinding sequence (grit size) was 120-240-320-400-600-800-1200 using SiC sandpaper. The polishing sequence (microns) was 6-3-1-0.5-0.25 using a glycol-based diamond suspension for the first 3 steps and a diamond paste with BlueLube polishing lubricant for the last 2 steps.

One of the 10 polished cross-sections is shown in Figure 7B. A sticker was applied to mark the edge of the substrate (Figure 7C). Using a Struers Duramin hardness tester with a load of 9.81 N (1 kgf) and holding time of 10 s, 5 Vickers hardness indentations were made along the build direction of each thin-wall cross-section, beginning near the substrate and moving outward, as shown in Figure 7D. A 0.5-mm approximate distance was held between the substrate and the first indentation to avoid hitting the 2 initial layers, which had used higher laser power. The indentations were spaced 0.5 mm apart, maintaining the required spacing of at least 2.5 times the diagonal length of each indentation as per ASTM E92-17 [52]. Additionally, in accordance with [52], a minimum distance of 2.5 times the Vickers diagonal length was maintained, where possible, between the indentation center and the sample edge. Although some deposits were tall enough to permit a 6th indentation, this point was discarded to maintain a consistent number of data points.

One deposit for 10% UHSLA (replicate 1, run 2) contained an unusually large pore, allowing space for only 3 indentations. Aside from this sample, all deposits contained 5 indentations that were used in the subsequent analyses. Five indentations × 10 deposits = 50 indentations per composition, giving about 500 total indentations. Out of this total, 5 indentations were too close to the sample’s outer edge to meet ASTM E92-17 [52]. Nevertheless, these measurements were included in the dataset, as they did not significantly deviate from the other measurements. In a few cases, the hardness measured at a particular point (designated Point X in Figure 7E) was unusually low compared to the 4 neighboring measurements or had a deformed shape, possibly indicating that the indenter had hit a pore, compositional inhomogeneity, or other defect. (Note the presence of entrapped gas porosity in Figure 7D,E [6,10].) In such cases, two additional measurements on either side were averaged and used in place of the reading at Point X.

Multiple linear regression (standard least squares) was performed on the Vickers hardness data using JMP Pro (version 16) statistical analysis software to assess each alloy’s hardness responses to the input factors. After hardness measurement, each sample was etched by dipping it in a 2% Nital solution for 10–15 s. Microstructures were viewed under an optical microscope (OM) and scanning electron microscope (SEM), specifically the Axia ChemiSEM by ThermoFisher Scientific (Waltham, MA, USA).

To check whether the in situ powder mixing strategy had produced the target compositions, energy dispersive spectroscopy (EDS) via SEM was used to estimate the average alloying element concentrations (wt%) in each sample. The primary purpose of these measurements was not to obtain accurate absolute readings, as standardless EDS analysis often suffers from systematic errors [53]. Rather, their purpose was to assess the trends across multiple alloys. An EDS line scan consisting of 3 points was conducted for each deposit, with each point located near one of the hardness indentations, as indicated by the blue “+” signs in Figure 7D. Three points per deposit × 10 deposits = 30 EDS readings per composition. Seven elements (Fe, C, Cr, Ni, Si, Mo, and Mn) were included in the analysis, corresponding to the primary elements in UHSLA steel. The collection time was 60 s per point using an acceleration voltage of 15 kV and spot size of 7.0–9.0, achieving an average count rate well above 100,000 counts/s for most points.

### 2.4. Experiment #2: Tensile Property Sensitivity

**Figure 8:**
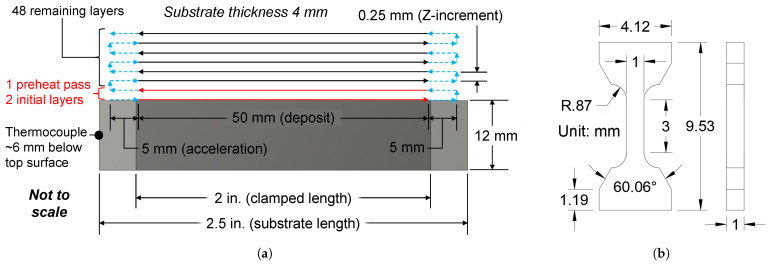
(**a**) Thin-wall deposition strategy and (**b**) MT2 tensile specimen dimensions.

After analyzing the results from Experiment #1, five compositions of interest were selected for a targeted analysis of tensile property sensitivities with respect to a single input factor. As discussed in Section 3.4 and Section 3.7, 70% UHSLA seemed to mark a transition in the hardness sensitivity. Choosing this alloy, along with compositions equidistant on either side, rendered the following selection: 40%, 50%, 70%, 90%, and 100% UHSLA. Interlayer delay was chosen as the input variable largely for practical reasons; maintaining relatively high laser power ensured the sample widths were sufficient to extract tensile specimens. Two thin-wall samples of each alloy were deposited using interlayer delay times of 1 s and 11 s. Figure 5 provides end- and side-view images of the setup. Figure 8a depicts the deposition strategy. A high laser power of 1100 W was used during the preheat pass and first 2 layers to ensure adequate bonding with the substrate. Forty-eight remaining layers were then deposited using a laser power of 900 W. The scan speed was 10 mm/s, and the total powder feed rate was 4.0 g/min, with the individual feed rates of the UHSLA and pure iron powders determining the composition. Argon carrier gas and shield gas were fed at 0.12 L/min through each powder line and 3.0 L/min around each nozzle, respectively.

The substrate material was 1018 low-carbon steel bar stock, 2.5 inches long × 12 mm tall × 4 mm thick. The middle 2-inch section was clamped between two 4340 steel blocks to help conduct heat away from the substrate. The deposit length was 50 mm, plus a 5 mm travel distance on each end of the deposit in which the laser was turned off while the robot accelerated and decelerated to move to the next layer; this ensured a constant scan speed within the deposit area. The Z-increment was 0.25 mm for each layer. Fifty layers × 0.25 mm gave a total height of about 12.5 mm, which varied slightly depending on the actual layer height. The wall thickness was about 2.5 mm. As shown in Figure 5B, a thermocouple was attached to one end of the substrate about 6 mm below the top surface to track the temperature during the deposition of the 70% UHSLA samples.

**Figure 9:**
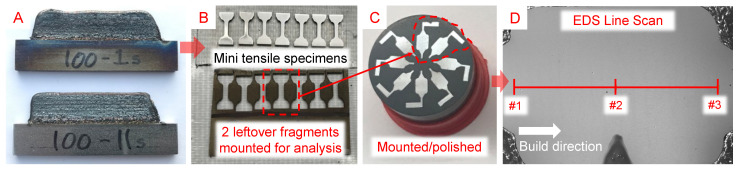
(**A**) Close-up view of two deposits, (**B**) EDM-cut mini tensile specimens, (**C**) mounted fragments for microstructure analysis, and (**D**) EDS line scan layout.

The completed experiments resulted in 10 samples, 5 for each composition × 2 interlayer delay times. Figure 9A shows the two deposits of 100% UHSLA. Seven miniature tensile (MT2) specimens [54] were cut out of each sample using a wire electrical discharge machine (EDM). Figure 8b gives the nominal specimen dimensions. After EDM cutting, the specimens were hand-polished using 600- followed by 800-grit SiC sandpaper. Figure 9B shows an example of the specimens, numbered 1–7 from left to right. Due to the hand-polishing process, the actual thickness was typically less than the nominal value of 1 mm. In order to accurately calculate tensile stresses, a point micrometer was used to obtain 3 measurements of the actual gauge thickness and width, which were averaged to obtain the cross-sectional area of each specimen.

Two leftover fragments between the cut-out specimens near the middle of each deposit were mounted, ground, polished to a 0.25-μm finish, and etched using the procedures described in Section 2.3. Figure 9C shows a few of these fragments, whose microstructures were analyzed via OM and SEM. EDS was used to roughly estimate the alloying element contents. A line scan of 3 points (Figure 9D) was run along the build height in the region corresponding to the gauge section of the tensile specimen. Three points per fragment × 2 fragments per deposit × 2 deposits per composition gave 12 total EDS readings per alloy. Fe, C, Cr, Ni, Si, Mo, and Mn were included in the analysis. The collection time was 60 s per point with an acceleration voltage of 15 kV and a spot size of 7.0, giving an average count rate well above 100,000 counts/second for most points.

Tensile testing of the MT2 specimens was conducted using a Tinius Olsen model 25ST with a 5-kN load cell. After applying a 90-N preload to remove the slack from the gripping assembly, the strain reading was zeroed before continuing the test. A crosshead position rate of 0.25 mm/min was used until reaching the 0.3% offset point; the speed was increased to 0.50 mm/min for the remainder of the test. An extensometer could not be attached to each specimen due to the very small gauge section, so the strains were estimated from the crosshead displacement. This led to inaccurate readings of elastic strain. Thus, the elongation values reported in this paper are based on stress–strain curves with reconstructed elastic regions. The modified curves assume an elastic modulus of 210 GPa (common for steels [55]) up to the elastic limit, which was approximated as the 0.2% offset yield strength from the original curve. Although the reported elongation and modulus of toughness values may contain slight errors due to the assumption of an elastic modulus, such errors are likely minimal since the vast majority of strain occurs in the plastic region. Note this modification did not affect the tensile and yield strength measurements, as these were based on the original, unaltered curves.

A few specimens which were used to tune the machine settings or failed during EDM cutting were not included in the final dataset. These specimens (%UHSLA/interlayer delay/specimen number) were 100/1/1, 100/1/2, 100/11/1, 90/1/1, and 50/11/3. Additionally, 50/1/7 was excluded as a probable outlier, which was supported by the Grubbs’ outlier test for the UTS and YS. Aside from these specimens, 7 stress–strain curves were collected from each deposit. Linear regression analysis (standard least squares) via JMP Pro (version 16) statistical software was performed on the tensile data to evaluate each alloy’s tensile property responses to interlayer delay time.

## 3. Results and Discussion

### 3.1. EDS Analysis

**Figure 10:**
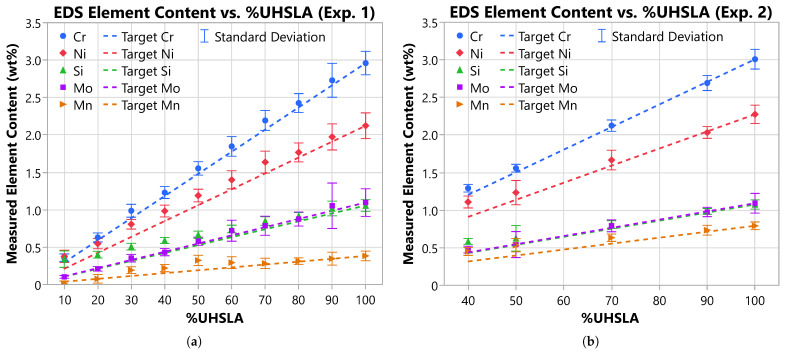
EDS measurements of alloying element contents: (**a**) Experiment #1 and (**b**) Experiment #2.

The EDS results for Experiment #1 (Exp. 1) and #2 (Exp. 2) are graphed in Figure 10. The plotted points represent the average measured concentrations (wt%) of Cr, Ni, Si, Mo, and Mn for each UHSLA/iron mixture, with error bars indicating the standard deviations. The error bars are generally small, indicating decent uniformity in composition. It is likely, however, that slight variations existed due to the nature of the in situ alloying process. The dashed lines represent the target concentrations, which were calculated relative to the EDS measurements for unmixed UHSLA. The target values follow a linear trend in direct proportion to the mass fraction of UHSLA contained in the mixture. For example, if the average Cr content was measured to be 3.0 wt% at 100% UHSLA, the target Cr content for a mixture containing 70% UHSLA was calculated as (70/100)×3.0wt%=2.1wt%. At 100% UHSLA, the target composition is equal to the measured composition since UHSLA serves as the reference point. Note the EDS measurements of the carbon content are not shown due to inaccuracies caused by organic contaminants on the sample surface.

As is evident in Figure 10, the measured concentrations are generally close to the target values, following linear trends with respect to %UHSLA. The measured Cr content tracks particularly well with the target. While the trends for Ni, Si, and Mo deviate slightly, they still show good linearity. The measured Mn content exhibits more variability, possibly because the quantity of this element is especially low, leading to more difficulty in obtaining an accurate reading. Note the average measured Ni content at 100% UHSLA (2.0–2.5 wt%) was significantly higher than that reported by the supplier (1.0 wt%), possibly due to EDS inaccuracies. Nevertheless, the trends suggest that the in situ mixing strategy produced approximately the target compositions relative to 100% UHSLA.

### 3.2. Temperature History

**Figure 11:**
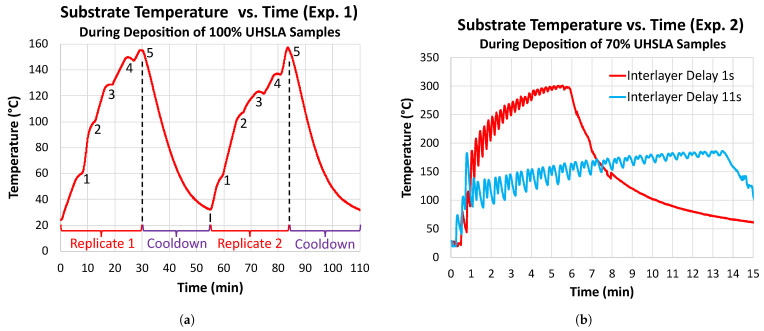
Substrate temperature histories: (**a**) Experiment #1 (hardness), recorded by pyrometer during deposition of 100% UHSLA samples. (**b**) Experiment #2 (tensile), measured by thermocouple during deposition of 70% UHSLA samples.

Figure 11a plots the temperature history recorded by the pyrometer at the clamped end of the substrate during the deposition of all 10 thin walls containing 100% UHSLA. While not necessarily representative of the thermal history near the deposition site, the plot conveys the bulk heat accumulation in the substrate. The two major peaks in temperature correspond to the two experimental replicates, as the substrate was allowed to cool before beginning replicate 2. During each replicate, the substrate temperature rose as samples were deposited, reaching a maximum of 150–160 °C at the clamped end. The five “bumps” within each of the two major peaks correspond to the five deposits within each replicate. It is worth noting that deposits added later within a replicate experienced higher starting substrate temperatures. A higher preheat temperature leads to a lower thermal gradient between the substrate and the deposit, resulting in a lower cooling rate and potentially a reduction in hardness [56]. Additionally, previously deposited thin walls might have undergone tempering due to reheating as subsequent deposits were added nearby. Thus, it is possible that the run order had an influence on the hardness as a consequence of depositing many samples on a single substrate.

Figure 11b plots the substrate temperature histories recorded by the thermocouple during the deposition of 70% UHSLA samples using interlayer delays of 1 s (blue) and 11 s (red). As is evident, the accumulation of heat was much greater for the shorter interlayer delay. While the end of the substrate reached a maximum temperature of less than 200 °C for the longer delay, it reached up to about 300 °C for the shorter delay. Greater heat build-up leads to lower temperature gradients and slower cooling rates [57,58], which in turn impacts the tensile properties, as discussed in subsequent sections.

### 3.3. Vickers Hardness

**Figure 12:**
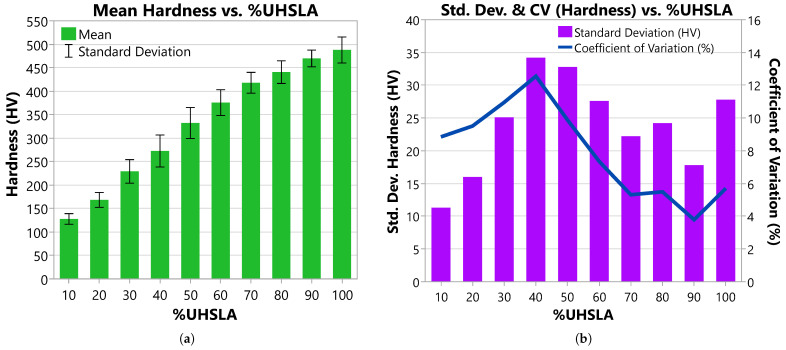
Hardness results including data from both replicates: (**a**) mean Vickers hardness and (**b**) standard deviation and coefficient of variation.

**Figure 13:**
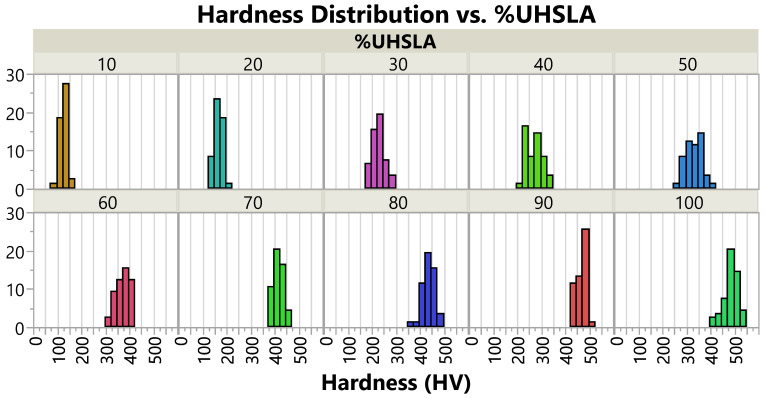
Vickers hardness distribution for each composition (both replicates included).

Figure 12 and Table 3 summarize the mean Vickers hardness, standard deviation (Std. Dev.), and coefficient of variation (CV) for each composition, with both replicates included in the calculations. Figure 13 gives histograms depicting the hardness distributions. The CV is equal to the standard deviation expressed as a percentage of the mean, representing variability relative to magnitude. For reference, Table 3 also includes approximate Rockwell C hardness (HRC) values, calculated using ASTM E140-12b equation A1.1.1 [59].

**Table 3:** Summary of hardness results (both replicates included in calculations).

%UHSLA	10	20	30	40	50	60	70	80	90	100
**Mean Hardness (HV)**	127.7	168.2	229.0	272.5	332.1	375.5	417.8	440.6	469.7	487.7
**Mean Hardness (HRC)** *	-	-	-	26	34	38	42	44	47	48
**Std. Dev. Hardness (HV)**	11.3	16.0	25.1	34.2	32.8	27.6	22.2	24.2	17.8	27.8
**CV Hardness (%)**	8.9	9.5	11.0	12.5	9.9	7.4	5.3	5.5	3.8	5.7

* Conversion to HRC only valid for ≥20 HRC [59].

As expected, the mean hardness increases with increasing %UHSLA (Figure 12a), from 128 HV at 10% UHSLA to 488 HV at 100% UHSLA, due to the increasing wt% of carbon and other alloying elements [37]. The standard deviation follows an interesting trend (bar chart in Figure 12b). Starting from 10% UHSLA, the standard deviation increases until reaching a maximum of 34.2 HV at 40% UHSLA. Beyond this point, the standard deviation decreases as more UHSLA is added, with the notable exception of a substantial peak at 100% UHSLA and a smaller peak at 80% UHSLA. Qualitatively, this trend can be observed in the histograms in Figure 13. The CV follows a similar pattern, reaching a peak of 12.5% at 40% UHSLA and then dropping off as more UHSLA is added (line graph in Figure 12b). Because the mean hardness decreases as more iron is added, the drop in the CV below 40% UHSLA is less pronounced than the corresponding drop in the standard deviation. The behavior of the standard deviation may suggest that the alloys containing 40–50% UHSLA are more sensitive to processing parameters.

### 3.4. Hardness Sensitivity

**Figure 14:**
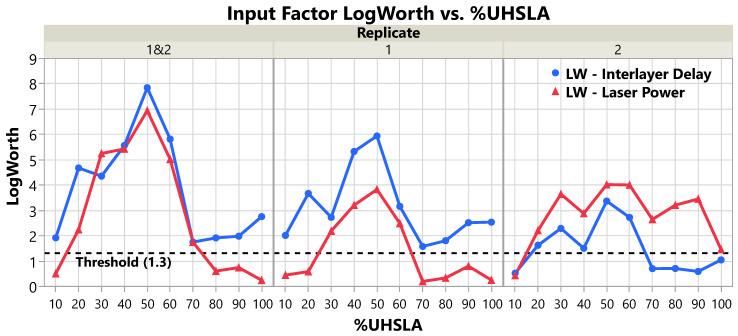
LogWorth of laser power and interlayer delay grouped by replicate(s) included.

**Figure 15:**
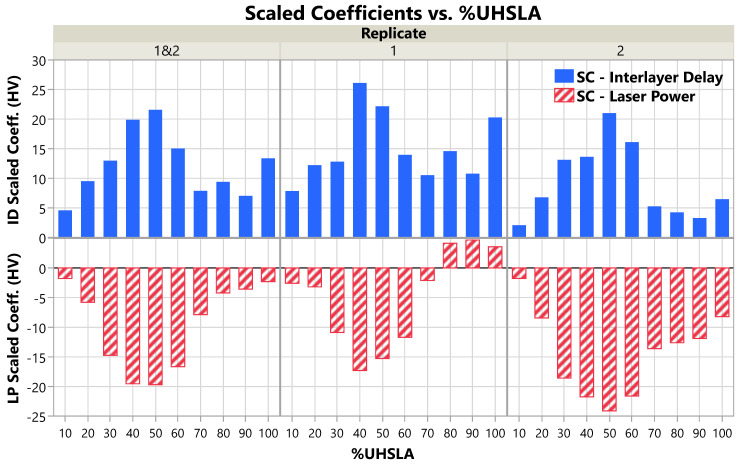
Scaled coefficients of laser power and interlayer delay grouped by replicate(s) included.

The R^2^, LogWorth (LW), and scaled coefficient (SC) values resulting from regression analysis are given in Table 4, Table 5, and Table 6, respectively. The LWs and SCs are additionally plotted in Figure 14 and Figure 15, respectively. Each table and figure is subdivided into three sections according to which replicates were included in the multiple regression model. Sections labeled “Replicate 1 and 2” contain results generated using both replicates together, while the other two sections represent each replicate separately. As discussed in Section 2.1, the LW indicates whether the laser power (LP) and interlayer delay (ID) had a statistically significant impact on the Vickers hardness. The SC indicates the amount of change in the hardness as an input travels half its experimental range.

With both replicates included in the regression model, alloys containing 30–60% UHSLA exhibit R^2^ values ranging from 0.50 to 0.65, while those containing 10–20% or 70–100% UHSLA show R^2^ ranging from 0.15 to 0.40 (Table 4). This indicates that the models are most effective from 30–60% UHSLA, outside of which the goodness of fit diminishes substantially. Replicates 1 and 2 follow similar patterns with some noteworthy differences. Compared to replicate 1, which has R^2^ peaking markedly at 40–50% UHSLA, replicate 2 shows more uniformity from 20–90% UHSLA. Nevertheless, both replicates generally agree that the models are most effective between 30% and 60% UHSLA.

**Table 4:** Coefficient of determination (R^2^) for each composition, grouped according to which replicates were included in the regression model. Higher R^2^ indicates a better fit to the data.

%UHSLA	10	20	30	40	50	60	70	80	90	100
**R^2^—Replicate 1&2**	0.15	0.40	0.50	0.54	0.65	0.54	0.21	0.15	0.16	0.20
**R^2^—Replicate 1**	0.30	0.49	0.50	0.70	0.75	0.55	0.21	0.25	0.38	0.35
**R^2^—Replicate 2**	0.08	0.41	0.57	0.46	0.64	0.61	0.38	0.45	0.47	0.27

In Figure 14, the dashed line marks the LogWorth threshold of 1.3, where LogWorth > 1.3 (*p*-value < 0.05) is required for a statistical significance level α=0.05 ([47], pp. 66–81). For alloys containing 30–60% UHSLA, there is strong evidence to suggest that laser power and interlayer delay influenced the hardness, as the LogWorth values are consistently above the threshold. For 10–20% and 70–100% UHSLA, the evidence is less strong, as the LogWorth sometimes falls below the significance threshold depending on which replicates are included in the regression model.

**Table 5:** Input factor LogWorth (LW) grouped according to which replicate(s) were included in the model. Higher LW indicates stronger statistical evidence that the input influenced the hardness.

	%UHSLA	10	20	30	40	50	60	70	80	90	100
**Replicate 1&2**	${\mathrm{LW}}_{\mathrm{LP}}$	0.51	2.24	5.25	5.43	6.94	5.02	1.75	0.61	0.74	0.25
	${\mathrm{LW}}_{\mathrm{ID}}$	1.92	4.68	4.36	5.57	7.85	5.82	1.75	1.92	1.98	2.76
**Replicate 1**	${\mathrm{LW}}_{\mathrm{LP}}$	0.45	0.59	2.19	3.21	3.83	2.49	0.20	0.34	0.80	0.25
	${\mathrm{LW}}_{\mathrm{ID}}$	2.01	3.67	2.73	5.33	5.94	3.16	1.58	1.81	2.52	2.54
**Replicate 2**	${\mathrm{LW}}_{\mathrm{LP}}$	0.43	2.21	3.65	2.88	4.02	4.01	2.65	3.21	3.45	1.46
	${\mathrm{LW}}_{\mathrm{ID}}$	0.52	1.63	2.29	1.51	3.37	2.73	0.71	0.71	0.59	1.05

The magnitudes of the scaled coefficients (Figure 15) follow a similar trend as the standard deviations from Figure 12b, which is to be expected if the input factors were a significant source of hardness variation. As is evident in Figure 15 with both replicates in the model (left plot), the hardness sensitivity with respect to both input factors peaks at 40–50% UHSLA. At 10% UHSLA, $|SC_{LP}|$ = 1.8 HV and $|SC_{ID}|$ = 4.6 HV. As the UHSLA content increases, $|SC_{LP}|$ and $|SC_{ID}|$ increase until reaching 19.7 HV and 21.6 HV, respectively, at 50% UHSLA. From 50% to 70% UHSLA, the response magnitudes drop dramatically. Above 70% UHSLA, the SCs hold fairly steady, with the exception of a noticeable increase in $|SC_{ID}|$ at 100% UHSLA. Even so, with $|SC_{LP}|$ = 2.3 HV and $|SC_{ID}|$ = 13.4 HV, 100% UHSLA exhibits a substantially lower hardness sensitivity than 40% and 50% UHSLA.

The SC plots for each individual replicate (Figure 15, middle and right) show similar trends with some noteworthy differences. In replicate 1, $|SC_{ID}|$ shows a particularly distinct peak at 100% UHSLA, although it is still smaller than those at 40–50% UHSLA. Compared to replicate 1, replicate 2 shows a sharper drop in response to interlayer delay as the UHSLA content increases above 60%. In replicate 2, $|SC_{ID}|$ remains very low from 70–100% UHSLA. In general, the hardness appears to have been more strongly influenced by interlayer delay in replicate 1 and by laser power in replicate 2.

**Table 6:** Input factor scaled coefficients (SCs) grouped according to which replicate(s) were included in the model. Higher SC indicates a steeper correlation between the hardness and the input.

	%UHSLA	10	20	30	40	50	60	70	80	90	100
**Replicate 1&2**	${\mathrm{SC}}_{\mathrm{LP}}$	−1.8	−5.8	−14.7	−19.5	−19.7	−16.7	−7.9	−4.2	−3.6	−2.3
	${\mathrm{SC}}_{\mathrm{ID}}$	4.6	9.5	13.0	19.9	21.6	15.0	7.9	9.4	7.0	13.4
**Replicate 1**	${\mathrm{SC}}_{\mathrm{LP}}$	−2.6	−3.2	−10.9	−17.3	−15.3	−11.7	−2.1	4.2	4.7	3.6
	${\mathrm{SC}}_{\mathrm{ID}}$	7.9	12.2	12.8	26.1	22.2	14.0	10.5	14.6	10.8	20.3
**Replicate 2**	${\mathrm{SC}}_{\mathrm{LP}}$	−1.8	−8.4	−18.6	−21.8	−24.1	−21.6	−13.6	−12.6	−11.9	−8.2
	${\mathrm{SC}}_{\mathrm{ID}}$	2.1	6.8	13.1	13.6	21.0	16.1	5.2	4.2	3.3	6.5

It is not entirely clear what caused the few discrepancies between the two replicates, although such discrepancies are not surprising due to the complexity of the L-DED process. There were undoubtedly additional noise factors which the regression models did not capture, such as defects in the powders, inhomogeneities from in situ mixing, or inconsistencies in the hardness measurement process. As discussed in Section 3.2, it is also possible that the experimental run order had an influence on the hardness. Some tempering might have occurred in samples from replicate 1 as those of replicate 2 were deposited nearby, contributing to the hardness variations in replicate 1.

The scaled coefficients are generally positive for interlayer delay and negative for laser power (Figure 15), i.e., increasing interlayer delay and decreasing laser power corresponded to an increase in hardness. This is largely explained by cooling rates. During solidification, the cooling rate near the melt pool is calculated as G×R, where *G* is the temperature gradient and *R* is the solid/liquid interface velocity ([60], p. 165). In AM, *R* is driven by the laser scan speed [2,6], which was held constant in this experiment. With a longer pause between layers, each layer has more time to cool. This increases the temperature gradient *G* between previous and new layers, which in turn increases the cooling rate [2,61]. Similarly, lower laser power leads to an increase in the cooling rate, as the surrounding material can more easily conduct heat away from the melt pool [6,8].

When a typical low-alloy steel undergoes equilibrium (slow) cooling from high-temperature austenite, the diffusion of carbon leads to the formation of ferrite or ferrite–pearlite ([37], pp. 59–100). Under rapid cooling, there is insufficient time for such diffusion to occur. Instead, carbon remains trapped in solution, forming the martensite phase ([37], p. 135). The lattice distortion caused by trapped carbon atoms leads to strain fields that resist dislocation movements, resulting in a strengthening effect that intensifies with increasing carbon content ([37], pp. 28, 29). At intermediate cooling rates, bainite transformation occurs, in which carbon precipitates into Fe_3_C carbides [37,62]. The strengthening effect of these carbides is much less pronounced than solid solution strengthening in martensite ([37], p. 196). Faster cooling also leads to finer grains [15] and higher dislocation densities [63], which contribute additional resistance to deformation. Note that replicate 1 shows hardness correlating positively with laser power for 80–100% UHSLA, which is the opposite of the normal trend. However, since the LogWorth values in this range were well below the significance threshold of 1.3, no firm conclusions can be drawn from this observation.

### 3.5. Tensile Properties

**Figure 16:**
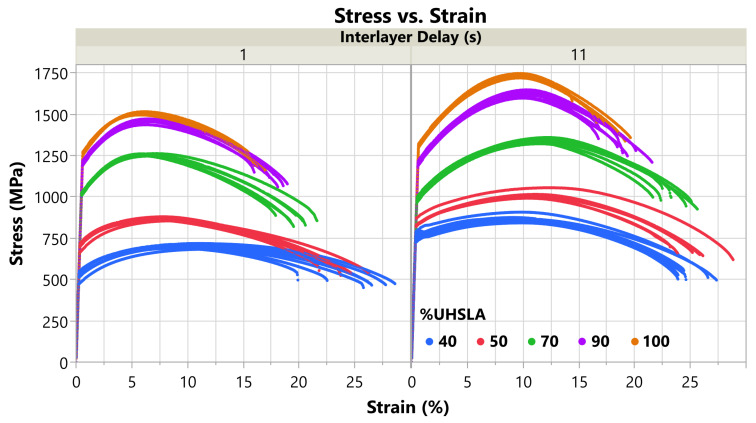
Stress–strain curves: all tensile results (with reconstructed elastic regions).

Figure 16 plots the stress–strain curves obtained for each of the five compositions selected for tensile analysis. Note the elastic regions were reconstructed as discussed in Section 2.4. The plot is subdivided vertically by interlayer delay time. Table 7 summarizes the average ultimate tensile strength (UTS), 0.2% offset yield strength (YS), elongation to fracture (%El), and modulus of toughness for each alloy, plus or minus (±) the standard deviation. The modulus of toughness is calculated as the area under the stress–strain curve, representing the energy absorbed until fracture. The average UTS correlates positively with the UHSLA content, increasing from 785 MPa at 40% UHSLA to 1632 MPa at 100% UHSLA, as would be expected due to the increasing percentage of carbon and other alloying elements. The average YS follows the same trend, increasing from 640 MPa at 40% UHSLA to 1288 MPa at 100% UHSLA. The average %El follows the opposite trend, decreasing from 25.2% at 40% UHSLA to 16.9% at 100% UHSLA. This is not surprising since higher strength is often accompanied by increased brittleness, i.e., lower ductility.

**Table 7:** Average tensile results ± standard deviation (UTS, YS, %El, and modulus of toughness).

Data Included	%UHSLA	UTS (MPa)	YS (MPa)	%El	Modulus of Toughness (J/m^3^)
All Data	40	784.8 ± 86.3 *	640.2 ± 123.3 *	25.2 ± 2.3	1.78 × 10^8^ ± 0.24 × 10^8^
	50	939.5 ± 76.2 *	765.9 ± 71.2 *	24.8 ± 1.8	2.10 × 10^8^ ± 0.30 × 10^8^
	70	1296.3 ± 44.2 *	997.3 ± 21.6 *	21.7 ± 2.3	2.54 × 10^8^ ± 0.36 × 10^8^
	90	1547.6 ± 86.9 *	1202.2 ± 10.6 *	18.4 ± 1.5	2.58 × 10^8^ ± 0.29 × 10^8^
	100	1632.3 ± 120.3 *	1288.1 ± 24.2 *	16.9 ± 1.4	2.52 × 10^8^ ± 0.32 × 10^8^
Interlayer Delay 1 s	40	703.3 ± 14.1	523.5 ± 26.6	25.3 ± 3.1	1.62 × 10^8^ ± 0.21 × 10^8^
	50	868.3 ± 10.1	700.0 ± 20.1	24.1 ± 1.5	1.86 × 10^8^ ± 0.11 × 10^8^
	70	1254.6 ± 5.5	1016.4 ± 5.6	19.8 ± 1.2	2.22 × 10^8^ ± 0.14 × 10^8^
	90	1458.8 ± 12.6	1200.3 ± 11.3	17.8 ± 1.0	2.36 × 10^8^ ± 0.13 × 10^8^
	100	1507.0 ± 7.8	1264.2 ± 7.9	16.3 ± 0.4	2.26 × 10^8^ ± 0.54 × 10^8^
Interlayer Delay 11 s	40	866.3 ± 20.8	756.9 ± 21.7	25.0 ± 1.4	1.95 × 10^8^ ± 0.12 × 10^8^
	50	1010.6 ± 23.0	831.7 ± 18.4	25.5 ± 2.0	2.33 × 10^8^ ± 0.22 × 10^8^
	70	1338.0 ± 11.7	978.3 ± 11.3	23.6 ± 1.5	2.85 × 10^8^ ± 0.18 × 10^8^
	90	1623.7 ± 18.2	1203.9 ± 10.5	18.9 ± 1.7	2.77 × 10^8^ ± 0.24 × 10^8^
	100	1736.7 ± 10.4	1308.0 ± 8.4	17.4 ± 1.8	2.73 × 10^8^ ± 0.28 × 10^8^

* Standard deviations marked with asterisks (*) reflect trends in the UTS and YS sensitivities to interlayer delay.

The upper section of Table 7 (after the “±”) summarizes the standard deviations including data from both interlayer delay times. The standard deviations in both the UTS and YS decrease as the UHSLA content increases from 40% to 70%. Beyond 70% UHSLA, the standard deviation of the UTS increases substantially, while that of the YS remains low. These behaviors reflect sensitivities to interlayer delay time, as discussed in the next section. The standard deviations in the %El do not follow a clear pattern, although they are slightly lower at 90–100% UHSLA. At a given interlayer delay (bottom two sections of Table 7), the standard deviations are low for UTS and YS, indicating good consistency. By contrast, the standard deviations in the %El are somewhat high relative to the average values, indicating a considerable amount of scatter in the elongation data.

As is evident in Table 7, the modulus of toughness remains fairly constant as the UHSLA content decreases from 100% to 70%, implying the increase in ductility compensates for the reduction in strength. The toughness drops when the UHSLA content decreases to 50% and 40%, i.e., the increase in ductility is no longer large enough to compensate for the decrease in strength. This trend holds true for both interlayer delays. Thus, 70% UHSLA appears to be the limit at which the alloying element content can be reduced to improve ductility without sacrificing tensile toughness. Charpy impact testing would be required for a more thorough assessment of toughness, particularly where high strain rates are concerned. This is an opportunity for future work.

### 3.6. Tensile Property Sensitivity

**Figure 17:**
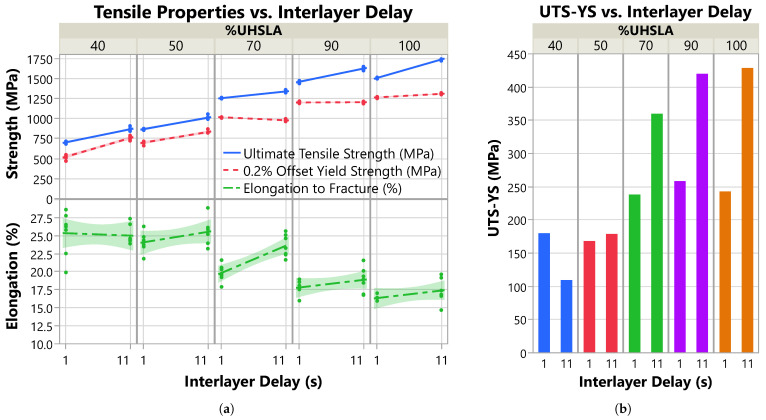
(**a**) Tensile properties vs. interlayer delay. (**b**) Ultimate tensile strength minus yield strength (representing work hardening) vs. interlayer delay.

Figure 17a plots the tensile data vs. interlayer delay for each of the five alloys, with best-fit lines obtained via linear regression. The regression results for each tensile property, including R^2^, LogWorth, and scaled coefficients, are listed in Table 8. For UTS, R^2^ is consistently ≥0.95, indicating the regression models fit the data well. The LogWorth of the UTS response ranges from 9.1–10.8, well above the significance threshold of 1.3 (*p*-value < 0.05). Thus, there is strong evidence suggesting that the interlayer delay influenced the UTS of all the alloys. The YS results are similar ($R^{2}\geq 0.84$, LogWorth≥5.0) with the exception of 90% UHSLA, whose YS response has an R^2^ and LogWorth of only 0.03 and 0.24, respectively, in which case the YS did not appreciably respond to interlayer delay.

The %El data exhibit much more scatter, evidenced by R^2^ and LogWorth values ≤0.17 and ≤0.74, respectively, for all alloys except 70% UHSLA (R^2^ 0.69, LogWorth 3.6). This scatter can be seen in the data points and 95% confidence bands shown in Figure 17a. Thus, with the exception of 70% UHSLA, there is insufficient evidence to conclude that the interlayer delay influenced the elongation.

**Table 8:** $R^{2}$, LogWorth ($LW_{ID}$), and scaled coefficient ($SC_{ID}$) for each tensile property.

%UHSLA	UTS- R^2^	YS- R^2^	%El- R^2^	UTS- ${\mathrm{LW}}_{\mathrm{ID}}$	YS- ${\mathrm{LW}}_{\mathrm{ID}}$	%El- ${\mathrm{LW}}_{\mathrm{ID}}$	UTS- ${\mathrm{SC}}_{\mathrm{ID}}$	YS- ${\mathrm{SC}}_{\mathrm{ID}}$	%El- ${\mathrm{SC}}_{\mathrm{ID}}$
40	0.96	0.96	0.01	9.08	9.32	0.10	81.49	116.70	−0.16
50	0.95	0.93	0.17	7.13	6.48	0.74	71.15	65.88	0.72
70	0.96	0.84	0.69	9.06	5.43	3.62	41.71	−19.09	1.88
90	0.97	0.03	0.14	8.95	0.24	0.67	82.44	1.76	0.53
100	1.00	0.90	0.15	10.79	5.00	0.61	114.83	21.90	0.52

**Figure 18:**
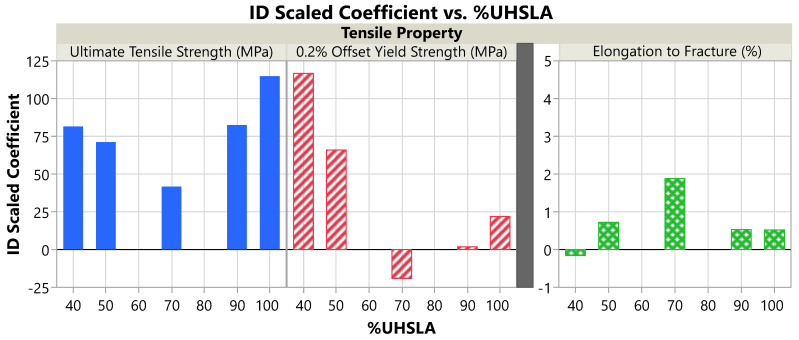
Influence of interlayer delay on the tensile properties: scaled coefficient vs. %UHSLA.

Corresponding to the slopes of the regression lines in Figure 17a, the scaled coefficients are plotted in Figure 18 and summarized in Table 8 (right). Specifically, the scaled coefficient is equal to the slope multiplied by 5 s, as discussed in Section 2.1. As the UHSLA content increases from 40% to 70%, the scaled coefficient for UTS decreases from 81.5 MPa to 41.7 MPa. Following a similar trend, the scaled coefficient for YS decreases sharply from 116.7 MPa to −19.1 MPa. Beyond 70% UHSLA, the UTS and YS behaviors diverge. While the scaled coefficient for UTS increases sharply to 114.8 MPa at 100% UHSLA, the YS sensitivity remains very low, only reaching 21.9 MPa at 100% UHSLA.

The divergence between the UTS and YS responses is related to work-hardening behaviors. Figure 17b plots the average difference between the UTS and YS vs. interlayer delay for each alloy. The quantity UTS–YS is indicative of the amount of work hardening undergone after yielding [64,65]. At 40% and 50% UHSLA, the work-hardening capability (UTS–YS) is relatively insensitive to interlayer delay. With UHSLA contents increasing to 70% and above, the work hardening capability becomes increasingly responsive.

As is evident in Figure 17a, the UTS correlates positively with interlayer delay time. As expounded in Section 3.4, increasing interlayer delay leads to steeper temperature gradients and higher cooling rates [2,58,61]. Faster cooling, in turn, leads to finer grains [15], higher dislocation density [63], and a higher probability that austenite will transform into martensite [37], all of which contribute to increased strength. With the exception of a slight negative slope at 70% UHSLA and a near-zero response at 90% UHSLA, the YS also trends upward with increasing interlayer delay time.

The %El appears to be most sensitive to interlayer delay at 70% UHSLA based on the scaled coefficients in Figure 18. Due to the high degree of scatter in the elongation data, however, the comparative magnitudes of the responses cannot be confidently assessed. At 70% UHSLA, the %El correlates positively with interlayer delay. This may be due to the faster cooling rate producing a finer grain structure, as grain refinement has been known to simultaneously improve strength and ductility [66,67]. It is also possible that the longer delay led to lower porosity, thereby improving the ductility.

### 3.7. Microstructure Analysis: Hardness Experiments

**Figure 19:**
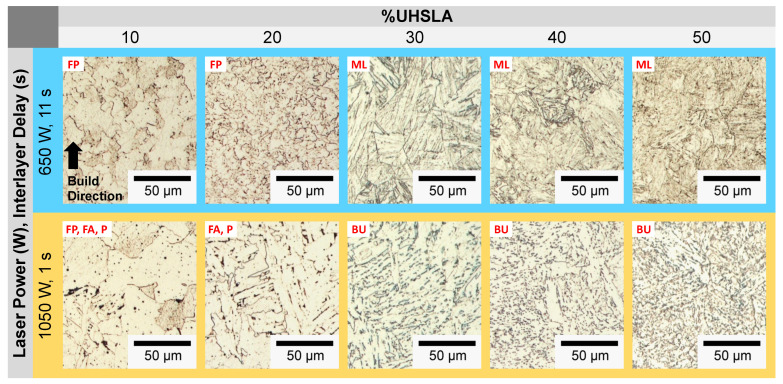
Optical micrographs: 10–50% UHSLA (replicate 2, near the 5th hardness indentation).

**Figure 20:**
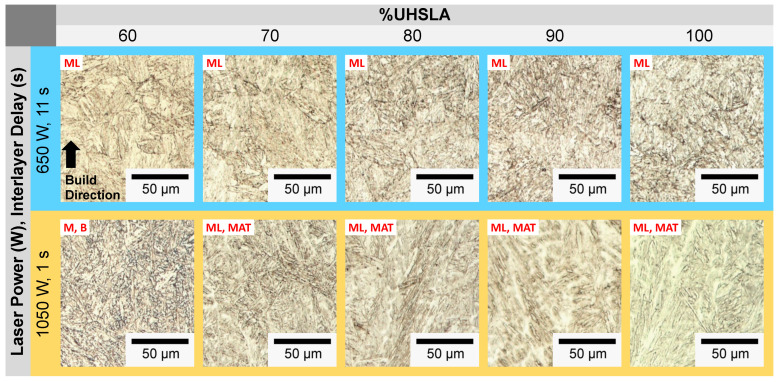
Optical micrographs: 60–100% UHSLA (replicate 2, near the 5th hardness indentation).

**Figure 21:**
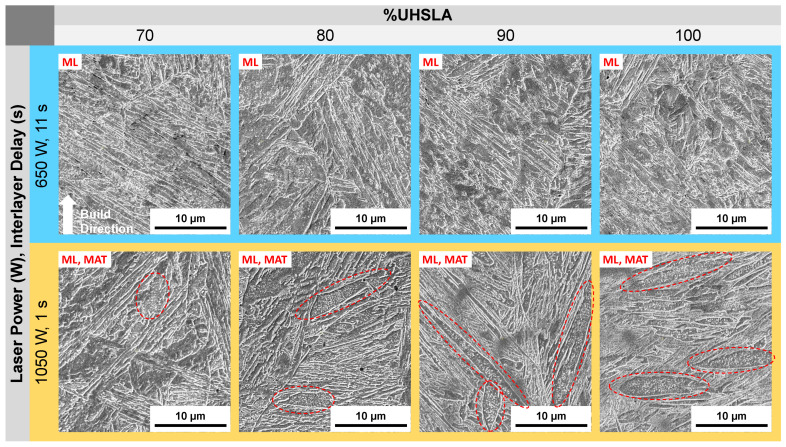
SEM micrographs: 70–100% UHSLA (circles highlight auto-tempered martensite).

The microstructural phases play a critical role in interpreting the hardness sensitivities. Figure 19 and Figure 20 provide optical micrographs for compositions containing 10–50% and 60–100% UHSLA, respectively. Each column corresponds to a different composition. The top row (blue) corresponds to a laser power of 650 W and interlayer delay of 11 s, while the bottom row (orange) corresponds to a laser power of 1050 W and interlayer delay of 1 s. As previously mentioned, lower laser powers and longer interlayer delays lead to higher cooling rates [2,8]. Hence, the top and bottom rows presumably represent the highest and lowest cooling rates, respectively, among the factor level combinations. Note all the images in Figure 19, Figure 20 and Figure 21 were taken from replicate 2 at locations near the 5th hardness indentation, i.e., roughly 2.5 mm from the substrate surface (refer to Figure 7D). Images from replicate 1 were generally in good agreement. Within each deposit, layers closer to the top exhibited larger prior-austenite grains due to the slower cooling rates farther from the substrate [8]; the phases were easier to distinguish in these regions.

Referring to Figure 19, the compositions containing 10% and 20% UHSLA exhibit ferritic microstructures. At higher cooling rates (top-row images), polygonal ferrite (FP) dominates. At lower cooling rates, some acicular ferrite (FA) can be observed, particularly for 20% UHSLA as evidenced by the elongated grain structure [68]; small amounts of pearlite (P) appear as black regions along grain boundaries. While the microstructural features differ in scale, with lower cooling rates producing larger grains, the consistent obtainment of ferrite explains the relatively low hardness variability of these alloys.

The compositions containing 30–50% UHSLA exhibit a stark contrast between their microstructures at higher vs. lower cooling rates. Samples undergoing higher cooling rates consist of primarily lath martensite (ML), while those subject to lower cooling rates consist of primarily upper bainite (BU) as evidenced by the Fe_3_C carbides (dark) precipitated between ferrite laths (light) and by the feathery appearance [62]. This result is consistent with the findings of Mendagaliev et al. in L-DED of the bainite-martensitic steel 09CrNi2MoCu, in which samples deposited with no interlayer delay contained higher amounts of bainite and ferrite (as opposed to martensite) compared to those experiencing a pause between layers [61]. This was attributed to the decrease in cooling rate arising from heat accumulation. Heat accumulation as a driver of bainite formation has been reported in other works on the laser deposition of low-alloy steels including 34CrNiMo6 [69], 300M [70], and 24CrNiMo [71]. Referring to Figure 20, 60% UHSLA shows similar behavior; while the higher cooling rate produces lath martensite, the lower cooling rate appears to produce a mixture of bainite (B) and martensite (M). Bainite is characteristically softer than martensite owing to the precipitation of carbon out of solution ([37], p. 196). Fluctuations between lath martensite and upper bainite may explain why the hardness sensitivity was high in alloys containing 30–60% UHSLA, especially at 40–50% UHSLA.

A significant change in the microstructural behavior occurs once the UHSLA content increases further, in that alloys containing 70–100% UHSLA exhibit lath martensite at both higher and lower cooling rates (Figure 20). The microstructures at faster cooling rates are more refined, with smaller prior-austenite grains and finer martensite laths. In a few of the images, a dendritic solidification structure can be observed. The avoidance of martensite/bainite fluctuations may be a key factor explaining the general reduction in hardness sensitivity at 10–20% and 70–100% UHSLA.

Figure 21 provides SEM images of 70–100% UHSLA at 5000× magnification. A key feature revealed by SEM, particularly noticeable from 80–100% UHSLA, is the presence of auto-tempered martensite (MAT) bands at lower cooling rates. Auto-tempering is known to occur in steels with relatively high martensite start (Ms) temperatures, typically low-alloy/low-carbon steels. In such steels, the first-formed martensite can be tempered during initial cooling if the cooling rate is sufficiently low [72]. The auto-tempered regions appear as dark packets containing small, white carbide precipitates (circled in Figure 21), while the untempered martensite laths appear smooth. Possible traces of auto-tempering can also be observed in 70% UHSLA at higher cooling rates. Auto-tempered martensite has been reported in the microstructures of low-alloy steels ER110S-G [73] and 300 M [74] deposited via WAAM. As with bainite, the precipitation of carbon renders auto-tempered martensite softer and weaker than lath martensite due to a loss of the solid solution strengthening effect ([37], p. 28). Thus, varying degrees of auto-tempering are a source of hardness sensitivity. With the possible exception of 100% UHSLA, which exhibited a relatively high standard deviation (see Figure 12b), the results seem to indicate that the detrimental impacts of martensite/bainite fluctuations on the robustness of as-built hardness are more severe than those arising from auto-tempering within the range of alloys studied.

### 3.8. Microstructure Analysis: Tensile Experiments

**Figure 22:**
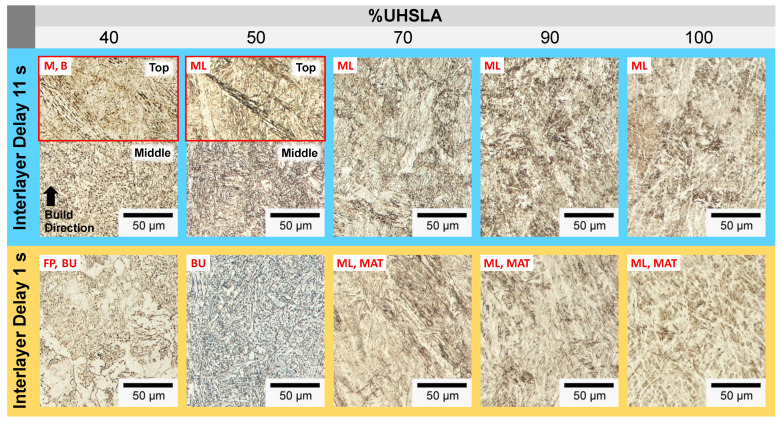
Optical micrographs: 40–100% UHSLA (middle of deposit height unless otherwise indicated by red outlines).

**Figure 23:**
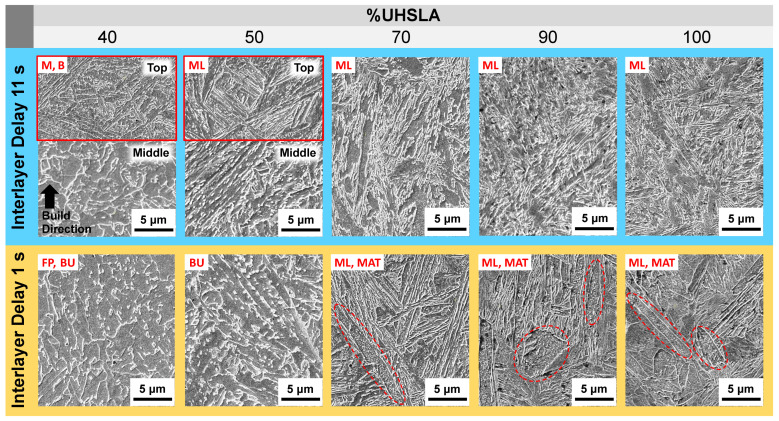
SEM micrographs: 40–100% UHSLA (middle of deposit height unless otherwise indicated by red outlines; circled regions highlight auto-tempered martensite).

Figure 22 and Figure 23 provide optical and SEM micrographs, respectively, of each alloy. All of the images were taken near the middle of the deposit height unless otherwise indicated. Additional images near the top of the deposit (outlined in red) are provided for 40% and 50% UHSLA to help clarify the microstructural behavior. Each column represents a different composition. The top and bottom rows represent interlayer delay times of 11 s (faster cooling rate) and 1 s (slower cooling rate), respectively.

In Figure 22 and Figure 23, the alloy containing 40% UHSLA appears to have produced a mixture of martensite and bainite near the top of the sample when deposited with an interlayer delay of 11 s. Near the middle of the deposit, the microstructure has a fine, granular appearance with carbides present. This may be indicative of an initial martensitic/bainitic structure which underwent recrystallization as a result of thermal cycling [75,76]. At the slower cooling rate, the 40% UHSLA microstructure contains a mixture of upper bainite (darker regions) and ferrite (lighter regions). The upper bainite is made evident by Fe_3_C carbides precipitated between ferrite laths [62].

The alloy containing 50% UHSLA produced primarily lath martensite at faster cooling rates and upper bainite at slower cooling rates, with little to no ferrite (Figure 22 and Figure 23). Similar to 40% UHSLA, the 50% UHSLA microstructure at the faster cooling rate appears to have undergone refinement near the middle of the deposit due to thermal cycling. The avoidance of the soft ferrite phase at slower cooling rates may have contributed to the reduction in YS sensitivity at 50% compared to 40% UHSLA (Figure 18).

As observed in the hardness experiments, a marked transition in the microstructure response occurs once the alloy content reaches 70% UHSLA. Rather than fluctuating between martensite and bainite, a lath martensite structure dominates at both cooling rates (Figure 22 and Figure 23). The avoidance of martensite/bainite fluctuations may be a key factor explaining the decrease in UTS and YS sensitivities at 70% UHSLA (Figure 18).

Once again, the SEM images of alloys containing 70–100% UHSLA reveal auto-tempered martensite bands (circled in Figure 23) prevalent at slower cooling rates. The visible quantity of auto-tempered martensite is significantly reduced at the faster cooling rate (i.e., longer interlayer delay).

As discussed previously, the YS remained relatively insensitive to interlayer delay at 90% and 100% UHSLA, while the UTS sensitivity increased sharply. This may reveal a particularly strong sensitivity of the work-hardening capability to auto-tempering at higher alloy contents. Such behavior is consistent with the findings of Tkachev et al., in which high-temperature tempering of a low-alloy 0.25C steel impacted the UTS more severely than the YS [77]. Mathevon et al. and Malheiros et al. demonstrated that higher-carbon steels in the as-quenched state generally have greater work-hardening potential than lower-carbon steels. Moreover, their results indicate that the effects of tempering on the work-hardening capability are more pronounced in higher-carbon steels [78,79]. Although these works focused on reheat-based tempering rather than auto-tempering, it is reasonable to suggest that auto-tempering produces similar effects.

### 3.9. Identifying an Optimal Composition

**Figure 24:**
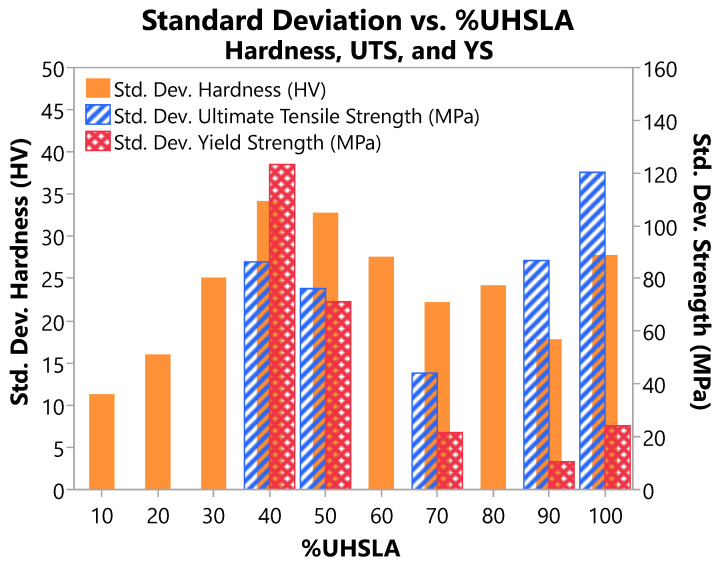
Comparative summary: standard deviations of mechanical properties vs. %UHSLA.

Figure 24 overlays the standard deviations of the Vickers hardness data (Table 3) with those of the UTS and YS data (Table 7). The standard deviations reflect sensitivities to the studied input factors and potentially other noise factors.

To identify an optimal alloy, robustness goals must be balanced with the desired performance. In this study, it is assumed that high hardness and strength are desired, along with good robustness against noise factors. Alloys containing 10–20% UHSLA exhibited good hardness robustness by consistently transforming to ferrite. However, they are unsuitable for high-strength applications. Alloys with 30–60% UHSLA suffered from martensite/bainite fluctuations, leading to poor robustness. At 100% UHSLA, the average hardness and strength were very high. However, the UTS was highly sensitive to interlayer delay, corresponding to a strong sensitivity of the work hardening capability.

At 70% UHSLA, the alloy content was just high enough to ensure transformation to martensite, rendering good robustness of hardness, YS, and UTS, as is evident in Figure 24. This was accompanied by relatively high averages for hardness (418 HV/42 HRC), strength (UTS 1296 MPa), and ductility (El 21.7%). While not as strong as UHSLA (UTS 1632 MPa), its improved ductility over UHSLA (El 16.9%) enabled it to maintain a modulus of toughness about equal to that of UHSLA. The 70% mixture represented the limit at which the UHSLA content could be reduced to improve ductility without sacrificing tensile toughness (Table 7). Considering the above factors, the alloy containing 70% UHSLA appears to exhibit a favorable combination of properties.

A few limitations of the present work should be noted. First, the sample size employed for tensile testing was relatively small. For a comprehensive assessment, further tests would need to be conducted, including an examination of impact toughness. Second, this study focused on a range of compositions within the category of low-alloy steels. As such, the results should not be construed as necessarily applying broadly to all steel varieties. Third, microstructural phases formed during initial cooling are not the only source of mechanical property sensitivity. Additional analysis techniques would be required to elucidate the sensitivities arising from grain size variations, tempering by cyclic reheating, residual stresses, entrapped gas porosity introduced by imperfections in the feedstock powders (Figure 3 and Figure 7D,E [6,10]), particle size distributions, etc. Finally, the cooling rates involved in deposition were not quantified directly. In situ monitoring of cooling rates would allow for a more direct evaluation of the microstructural responses.

## 4. Conclusions

This work investigated the effect of steel alloy composition on mechanical property robustness in L-DED when exposed to process variations. In situ blending of UHSLA steel and pure iron powders produced 10 compositions ranging from 10% to 100% UHSLA by mass. Vickers hardness sensitivities were analyzed via multiple linear regression with respect to laser power and interlayer delay time. In a subsequent experiment, tensile property sensitivities of five select alloys were analyzed with respect to interlayer delay. Microstructures aided the interpretation of the results. Key conclusions are as follows:

1. The hardness, ultimate tensile strength (UTS), and yield strength (YS) of alloys containing 40–50% UHSLA were highly sensitive to process variations, corresponding to cooling rate-driven phase fluctuations between lath martensite and upper bainite.
2. Alloys with low UHSLA contents (10–20%) transformed primarily to ferrite; those with high contents (70–100%) transformed to martensite, intermixed with auto-tempered martensite at lower cooling rates. The avoidance of martensite/bainite fluctuations appears to be a key factor leading to improved robustness.
3. At 70% UHSLA, the hardness, UTS, and YS were relatively robust, as the alloy content was just high enough to ensure transformation to martensite or auto-tempered martensite. This alloy maintained a relatively high hardness and strength while exhibiting superior ductility to UHSLA without sacrificing tensile toughness.
4. Above 70% UHSLA, the YS sensitivity remained low. In contrast, the UTS sensitivity increased, possibly suggesting a strong response of the work hardening capability to auto-tempering at higher alloy contents.
5. While hardness analysis captures certain microstructural sensitivities, it may not reveal other sensitivities relevant to the tensile properties, such as factors influencing the work hardening capability. This is an important consideration when designing experiments to evaluate the robustness of alloys.

EDS analysis indicated the in situ mixing strategy produced approximately the target compositions. The novel rotary deposition scheme enabled rapid collection of hardness data before conducting a more targeted tensile property analysis. A potential drawback to this setup is that it may allow previous samples to undergo tempering as new samples are deposited nearby. On the other hand, this feature could be leveraged to study the effects of additional noise factors. An opportunity for future research is to expand the in situ alloying setup to allow each element’s fraction to be tuned independently. Robustness evaluations could be extended into other material categories such as aluminum and titanium alloys. This work represents a step toward incorporating robust design strategies into the development of alloys for metal additive manufacturing.

## Data Availability

The data presented in this study are available on request from the corresponding author subject to approval from the Army Research Office.
